# Species distribution, antimicrobial resistance, and putative virulence factor profiles of *Aeromonas* isolates from extra-intestinal clinical specimens: a whole-genome sequencing study

**DOI:** 10.3389/fmicb.2026.1952096

**Published:** 2026-09-23

**Authors:** Qifeng Jiang, Xingbei Weng

**Affiliations:** Department of Clinical Laboratory, The First Affiliated Hospital of Ningbo University, Ningbo, Zhejiang, China

**Keywords:** *Aeromonas*, antimicrobial resistance, *bla_NDM-1_*, genotype–phenotype concordance, putative virulence factors, whole-genome sequencing

## Abstract

**Introduction:**

*Aeromonas* spp. are increasingly recognized opportunistic pathogens of humans, yet integrated genomic and phenotypic data from clinical, extra-intestinal isolates remain limited. We characterized 74 non-duplicate *Aeromonas* isolates recovered from extra-intestinal clinical specimens at a tertiary hospital in Ningbo, China, between 2020 and 2025, combining whole-genome sequencing (WGS)-based taxonomy, antimicrobial susceptibility testing, and resistome and putative virulence factor (VF) prediction.

**Methods:**

Species were identified by average nucleotide identity (ANI) analysis and sequence types (STs) by multilocus sequence typing (MLST). Susceptibility to 11 agents was interpreted according to the Clinical and Laboratory Standards Institute (CLSI) guideline M45. Resistance genes were predicted against the Comprehensive Antibiotic Resistance Database (CARD) and VFs against the Virulence Factors of Pathogenic Bacteria database (VFDB); genotype–phenotype concordance was quantified with Cohen’s κ, and VF profiles were analyzed by principal coordinates analysis and permutational multivariate analysis of variance (PERMANOVA).

**Results:**

Six species were identified, dominated by *Aeromonas caviae* (32.4%), *Aeromonas veronii* (25.7%) and *Aeromonas dhakensis* (24.3%). Bile was the leading source of isolation (51.4%), and species were significantly associated with isolation site (Fisher’s exact test, *p* < 0.001). The population was genetically diverse (71 STs, 39 novel). Resistance was highest to piperacillin/tazobactam (29.7%), whereas only one isolate was resistant to amikacin. Eighty-five resistance genes were detected, with a ubiquitous intrinsic β-lactamase background (*OXA-12-like*, *cphA/imiH* and *MOX-type* genes in 63.5, 58.1 and 41.9% of isolates, respectively) and the acquired carbapenemase gene *bla_NDM-1_* in three isolates. Genotype–phenotype agreement was substantial for trimethoprim/sulfamethoxazole (κ = 0.773–0.856) and fluoroquinolones (*κ* = 0.706) but poor for β-lactams. Fifty-six VFs were detected, comprising a conserved 26-VF core virulome and accessory determinants structured by species (PERMANOVA *R*^2^ = 0.797, *p* = 0.001). *A. dhakensis* and *A. hydrophila* carried the heaviest VF loads and were enriched for aerolysin, ExoA, MARTX/RtxA and T6SS, whereas *A. caviae* lacked aerolysin and had the sparsest virulome.

**Discussion:**

Species identity was the dominant correlate of clinical distribution and virulence potential, supporting genome-based species identification as the basis for risk assessment of clinical *Aeromonas* isolates.

## Introduction

The genus *Aeromonas* comprises Gram-negative, facultatively anaerobic bacilli that are ubiquitous in aquatic environments and are increasingly recognized as opportunistic pathogens of humans. In addition to gastroenteritis, aeromonads cause a wide spectrum of extra-intestinal infections, including biliary tract infection, bacteremia, respiratory and urinary tract infections, and wound infection, particularly in elderly or immunocompromised patients (Janda and Abbott, 2010; Parker and Shaw, 2011; Fernández-Bravo and Figueras, 2020). The taxonomy of the genus is complex and continues to evolve, and routine phenotypic identification frequently misclassifies clinically important species. In particular, *Aeromonas dhakensis*—first described in 2002—is commonly misidentified as *Aeromonas hydrophila* by conventional methods, yet comparative studies based on molecular identification have shown it to be both more prevalent and more virulent than previously appreciated (Chen et al., 2014a; Chen et al., 2016). Genome-based approaches such as average nucleotide identity (ANI) analysis are therefore increasingly required to resolve the species-level epidemiology of clinical aeromonads (Lu et al., 2025).

Antimicrobial resistance in *Aeromonas* is mechanistically distinctive. Aeromonads constitutively or inducibly produce up to three chromosomal β-lactamases—a penicillinase, an inducible cephalosporinase (AmpC) and a metallo-β-lactamase of the CphA/ImiH family—whose coordinated expression confers resistance to most β-lactams except fourth-generation cephalosporins and, usually, carbapenems (Rossolini et al., 1996). Superimposed on this intrinsic background, acquired resistance determinants, including extended-spectrum β-lactamases and carbapenemases such as NDM-1, have begun to emerge in the genus, raising concern because aeromonads occupy aquatic niches that interface directly with human activity (Singh et al., 2026). Whole-genome sequencing (WGS) combined with curated resources such as the Comprehensive Antibiotic Resistance Database (CARD) enables rapid resistome prediction (McArthur et al., 2013; Alcock et al., 2023), but the predictive value of genotypic screening is known to vary widely among organisms and drug classes and has rarely been evaluated systematically in *Aeromonas* (Luo et al., 2022; Wu et al., 2023; Pineda-Reyes et al., 2024).

The pathogenicity of aeromonads is likewise multifactorial, involving pore-forming toxins such as aerolysin, secreted enzymes, secretion systems (T2SS, T3SS, T6SS), adhesins and iron-acquisition systems (Tomás, 2012). Comparative and population genomic analyses indicate that virulence-gene repertoires are strongly species-structured, which may partly explain the divergent clinical behavior of the major species (Zhong et al., 2019; Abdella et al., 2023; Singh et al., 2026). However, few studies have jointly examined species distribution, population structure, antimicrobial resistance and virulence determinants in a single, well-characterized clinical collection.

Here, we analyzed 74 non-duplicate *Aeromonas* isolates consecutively recovered from extra-intestinal clinical specimens at a Chinese tertiary hospital between 2020 and 2025. Species were identified by WGS-based ANI analysis and validated by core-genome phylogenomics; phenotypic susceptibility to 11 agents was correlated with CARD-predicted resistomes; and VFDB-predicted virulomes were compared across species and isolation sources. Our aims were (i) to delineate the species spectrum and genetic diversity of clinical *Aeromonas* in this setting, (ii) to quantify the concordance between resistance genotypes and phenotypes across drug classes, and (iii) to define the species-level stratification of virulence potential, thereby providing an integrated framework for the clinical risk assessment of this increasingly important pathogen.

## Materials and methods

### Study design, bacterial isolates and clinical data collection

This retrospective study included 74 non-duplicate *Aeromonas* isolates consecutively recovered from extra-intestinal clinical specimens at the First Affiliated Hospital of Ningbo University (Ningbo, Zhejiang, China) between 2020 and 2025. Only the first isolate from each patient was included to avoid duplication. Isolates were obtained by the clinical microbiology laboratory following standard operating procedures, preliminarily identified by matrix-assisted laser desorption/ionization time-of-flight mass spectrometry (MALDI-TOF MS) using the VITEK MS system with the IVD knowledge base V3.2 (bioMérieux, Marcy-l’Étoile, France), purified and stored at −80 °C until further analysis. For every isolate, the specimen source (bile, blood, sputum, urine or wound pus) and the patient’s gender and age were retrieved from the laboratory information system. Species identification was confirmed by WGS-based ANI analysis, and sequence types (STs) were assigned according to the *Aeromonas* multilocus sequence typing (MLST) scheme (Martino et al., 2011).

### Antimicrobial susceptibility testing

Susceptibility of the 74 isolates to 11 agents belonging to seven classes was determined with the VITEK 2 automated system (bioMérieux, Marcy-l’Étoile, France) on the basis of the broth microdilution method, and results were interpreted as susceptible (S), intermediate (I) or resistant (R) according to the Clinical and Laboratory Standards Institute (CLSI) guideline M45 for *Aeromonas* spp. (Clinical and Laboratory Standards Institute [CLSI], 2016). The panel comprised piperacillin/tazobactam (TZP), ceftazidime (CAZ), cefoperazone/sulbactam (SCF), cefepime (FEP), aztreonam (ATM), imipenem (IPM), meropenem (MEM), ciprofloxacin (CIP), levofloxacin (LEV), amikacin (AMK) and trimethoprim/sulfamethoxazole (SXT). *Escherichia coli* ATCC 25922 and *Pseudomonas aeruginosa* ATCC 27853 served as the quality-control strains.

### Whole-genome sequencing and species identification

Genomic DNA was extracted from overnight cultures using an STE buffer-based phenol–chloroform method. Briefly, bacterial pellets (approximately 10 mg wet weight) were resuspended in 600 μL STE buffer, digested with 60 μL lysozyme at 37 °C for 30 min and then with 100 μL 10% SDS and 10 μL proteinase K at 65 °C for 20–30 min. The lysates were extracted successively with equal volumes of phenol:chloroform:isoamyl alcohol (25:24:1) and chloroform:isoamyl alcohol (24:1), and DNA was precipitated with 0.75 volumes of pre-chilled isopropanol at −20 °C for 30 min, washed twice with 75% ethanol, air-dried and dissolved in 50–100 μL elution buffer (EB) with 2 μL RNase, followed by incubation at 37 °C for 25 min to remove residual RNA. Sequencing libraries were prepared using the Rapid Plus DNA Lib Prep Kit for Illumina (cat. no. RK20208; ABclonal Technology, Wuhan, China) according to the manufacturer’s instructions and sequenced on the Illumina NovaSeq X Plus platform (Illumina, San Diego, CA, United States) with 150-bp paired-end reads. Raw reads were quality-filtered with fastp (Chen et al., 2018) and *de novo* assembled with SPAdes (Bankevich et al., 2012). Species identification was performed by ANI analysis using JSpeciesWS (Richter et al., 2016), comparing each assembled genome with reference genomes of *Aeromonas* type strains; an ANI value ≥ 95% was taken as the threshold for species assignment. For each isolate, the preliminary MALDI-TOF MS identification was retrieved from the laboratory information system and subsequently compared with the ANI-based species assignment.

### MLST and core-genome phylogenetic analysis

The STs were assigned *in silico* from the assemblies according to the *Aeromonas* MLST scheme (Martino et al., 2011) hosted on PubMLST (Jolley and Maiden, 2010); novel STs were registered in the PubMLST database. For phylogenetic analysis, core-genome SNPs of the 74 study isolates together with six NCBI reference genomes were identified using Snippy (v4.6.0) (Seemann, 2015), which produced a core-genome multiple-sequence alignment of variant sites. A maximum-likelihood phylogenetic tree was inferred with IQ-TREE (v2.4.0) (Minh et al., 2020) and visualized and annotated with iTOL v6 (Letunic and Bork, 2024). The six reference genomes were *A. caviae* GCF_900476005.1, *A. dhakensis* GCF_002285935.1, *A. hydrophila* GCF_000014805.1, *A. media* GCF_020423125.1, *A. trota* GCF_020097295.1 and *A. veronii* GCF_024971835.1.

### Prediction of antimicrobial resistance genes

Putative antimicrobial resistance genes (ARGs) were identified by querying the assemblies against CARD (McArthur et al., 2013; Alcock et al., 2023) using the Resistance Gene Identifier (RGI) tool (v6.0.8). Only hits with an amino-acid identity ≥ 80% and a query coverage ≥ 90% were retained, and duplicated hits of the same gene within the same isolate were collapsed into a single record. The retained ARGs were classified by drug class, gene family and resistance mechanism according to the CARD Antibiotic Resistance Ontology (ARO).

### Prediction of virulence factors

The 74 genome assemblies were annotated with Prokka (v1.12) (Seemann, 2014), and the predicted protein sequences were screened against the core dataset (Set A) of VFDB (Liu et al., 2022) using DIAMOND v2.2.2 in blastp mode (Buchfink et al., 2021); alignments with an amino-acid identity ≥ 60% and an E-value < 1 × 10^−5^ were retained. Using the VF name as the analytical unit, a presence/absence (0/1) matrix of putative virulence factors (VFs) across isolates was constructed, yielding 56 detected VFs. The 20 variable VFs present in 2–99% of the isolates were retained for downstream analyses.

### Statistical analysis

Categorical variables are summarized as numbers (percentages); age is presented as median and interquartile range (IQR) because of its non-normal distribution. Fisher’s exact test with a Monte Carlo–simulated *p*-value (100,000 replicates) was used as the primary analysis, with the Pearson chi-square statistic reported for reference; standardized residuals were calculated to identify the species–source combinations contributing most to the overall association, and an absolute residual > 2 was considered indicative of significant enrichment or depletion.

Agreement between resistance genotypes (carriage of ≥ 1 ARG annotated to the corresponding drug class) and phenotypes (resistance to any agent of the corresponding class) was assessed using contingency tables; sensitivity, specificity, positive and negative predictive values, and overall agreement were calculated with the phenotype as the reference. For these analyses, the phenotype was dichotomized by scoring resistant (R) isolates as positive, whereas intermediate (I) isolates were grouped with susceptible (S) isolates. Associations were tested by two-sided Fisher’s exact test, and the strength of agreement was quantified by Cohen’s *κ* coefficient (Cohen, 1960), interpreted as poor (<0.20), fair (0.21–0.40), moderate (0.41–0.60), substantial (0.61–0.80) and almost perfect (>0.80). The ubiquitously carried RND efflux pump gene *rsmA* was excluded from genotype calling because it was detected in all isolates and carried no discriminative information.

For VF analyses, pairwise Jaccard distances between isolates were computed from the variable-VF matrix using the vegan package (v2.7–5). A presence/absence heatmap was generated with pheatmap (v1.0.13), with isolates clustered by UPGMA (average linkage) on Jaccard distances and annotated by species and isolation source. Principal coordinates analysis (PCoA; classical multidimensional scaling, cmdscale) was performed on the Jaccard distance matrix and plotted with ggplot2 (v4.0.3); 95% confidence ellipses were drawn for groups with ≥ 3 isolates. Overall differences in VF profiles among species and among isolation sources were tested by permutational multivariate analysis of variance (PERMANOVA, vegan:adonis2, 999 permutations), and homogeneity of within-group dispersions was assessed with betadisper (999 permutations); when the global test was significant, pairwise PERMANOVA was performed with Benjamini–Hochberg (BH) adjustment. Differences in the carriage rate of each variable VF among species or sources were tested by Fisher’s exact test (Monte Carlo simulation, *B* = 10,000, for sparse tables) with BH correction. VF load was compared among groups by the Kruskal–Wallis test, followed by pairwise Wilcoxon rank-sum tests with BH adjustment when significant.

All analyses were performed in R v4.5.2 (R Foundation for Statistical Computing, Vienna, Austria). All tests were two-sided, and an (adjusted) *p*-value < 0.05 was considered statistically significant.

### Analysis of the *bla_NDM-1_* genetic context

For the three *bla_NDM-1_*-positive isolates, the *bla_NDM-1_*-carrying contigs were identified by BLASTN searches (Altschul et al., 1990) of the assemblies against the *bla_NDM-1_* reference sequence (GenBank accession no. NG_049326.1) with default parameters. Flanking regions were annotated by BLASTN alignment against the Tn125 module reference (JN872328.1; nucleotides 3,900–14,250), the IS26 element (NZ_JBLKGN010000080.1) and the PlasmidFinder replicon database (accessed July 2026); hits with ≥90% nucleotide identity were considered present, and weaker matches (≥75% identity) were reported as ‘-like’ elements. The 197-bp region immediately upstream of the *bla_NDM-1_* start codon was compared among the three isolates.

## Results

### Demographic and clinical characteristics of the study cohort

The 74 *Aeromonas* isolates were obtained from 51 male (68.9%) and 23 female (31.1%) patients, corresponding to a male-to-female ratio of 2.2:1. The median age of the patients was 69 years (IQR 60–76 years; range 17–92 years), and 56 patients (75.7%) were aged 60 years or older, indicating that the elderly constituted the predominant affected population. Bile was the most frequent source of isolation (*n* = 38, 51.4%), followed by sputum (*n* = 11, 14.9%), wound pus (*n* = 11, 14.9%), urine (*n* = 8, 10.8%) and blood (*n* = 6, 8.1%) (Supplementary Table S1).

### Species identification, MLST and core-genome phylogeny

The ANI analysis resolved the 74 isolates into six species: *A. caviae* (*n* = 24, 32.4%), *A. veronii* (*n* = 19, 25.7%), *A. dhakensis* (*n* = 18, 24.3%), *A. hydrophila* (*n* = 10, 13.5%), *A. media* (*n* = 2, 2.7%) and *A. trota* (*n* = 1, 1.4%). MLST assigned the 74 isolates to 71 distinct STs: 35 isolates belonged to known STs, whereas 39 isolates represented novel STs that were registered in the PubMLST database (Supplementary Table S1). The maximum-likelihood phylogeny based on core-genome SNPs resolved the 74 isolates into six well-defined clades that were fully concordant with the ANI-based species assignments, and the six NCBI reference genomes were placed within their corresponding species clades (Figure 1). Species-level identification by MALDI-TOF MS agreed with the ANI-based assignment for only 33 of the 74 isolates (44.6%). Concordance was high for *A. hydrophila* (10/10, 100%) and *A. caviae* (18/24, 75.0%) but poor for *A. veronii* (4/19, 21.1%); none of the 18 *A. dhakensis* isolates was correctly identified—nine (50.0%) were reported as *A. hydrophila*, three as *A. sobria*, three as *A. caviae*, two as *A. jandaei* and one as *A. veronii* (Supplementary Table S1). This pattern is consistent with recent direct comparisons of MALDI-TOF MS and genome-based identification in this genus (Vieira et al., 2025).

**Figure 1:**
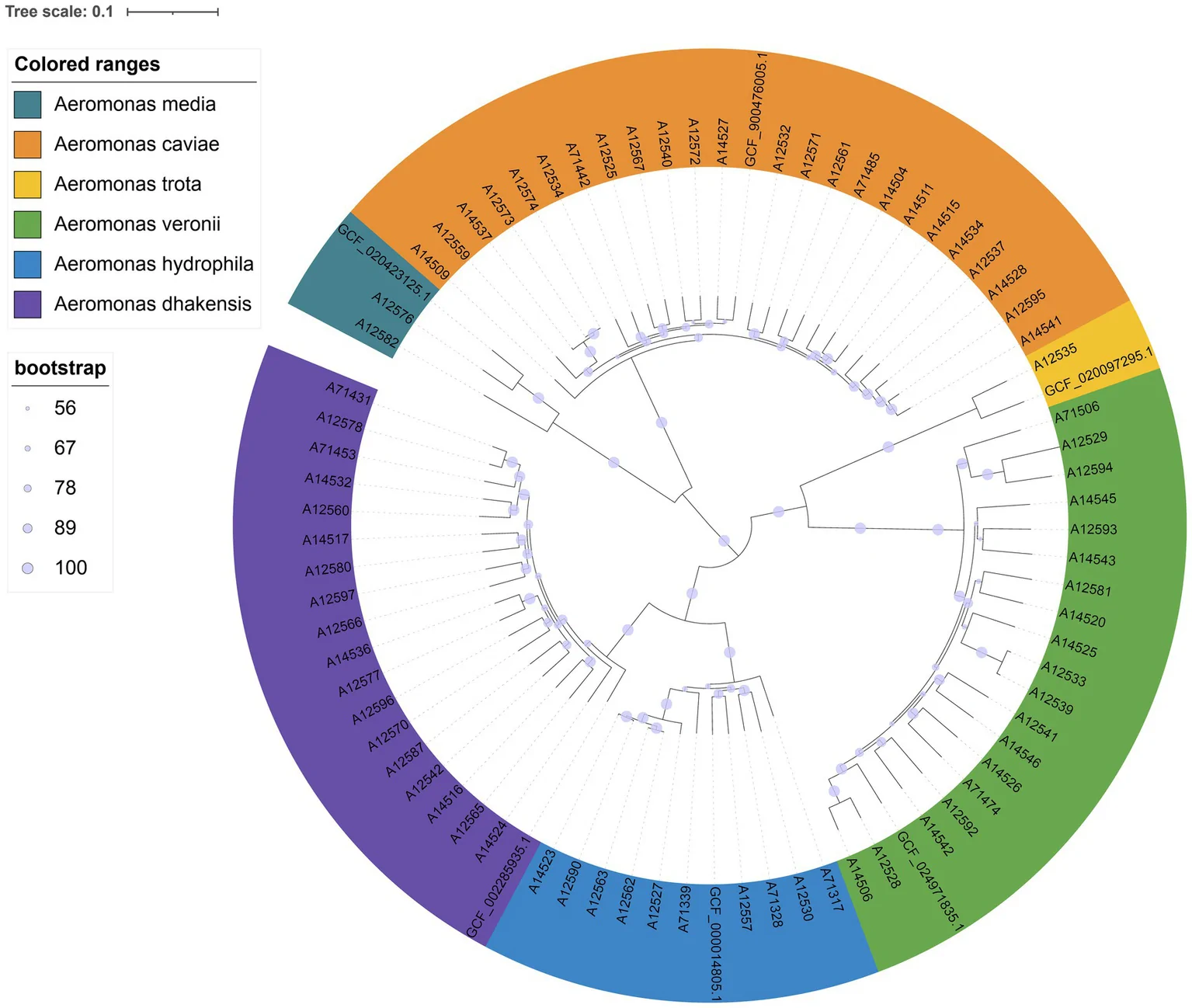
Maximum-likelihood phylogenetic tree of the 74 study isolates and six NCBI reference genomes based on core-genome SNPs. The tree was inferred with IQ-TREE and visualized with iTOL v6. Isolates are colored by ANI-defined species; the six species form six well-supported, mutually concordant clades.

### Distribution of *Aeromonas* species across isolation sources

The distribution of the six species across the five isolation sources is shown in Table 1 and Figure 2. Bile was the dominant source for the three most common species, accounting for 45.8% (11/24) of *A. caviae*, 61.1% (11/18) of *A. dhakensis* and 63.2% (12/19) of *A. veronii* isolates. In contrast, *A. caviae* was also frequently recovered from sputum (9/24, 37.5%), *A. hydrophila* showed a relative enrichment in urine (4/10, 40.0%), and *A. dhakensis* was the most common species among wound pus isolates (5/11, 45.5%).

**Table 1:** Distribution of *Aeromonas* species across isolation sources, *n* (%) within species.

Species	Bile	Blood	Sputum	Urine	Wound pus	Total
*A. caviae* (*n* = 24)	11 (45.8)	1 (4.2)	9 (37.5)	3 (12.5)	0 (0.0)	24 (100)
*A. veronii* (*n* = 19)	12 (63.2)	3 (15.8)	0 (0.0)	0 (0.0)	4 (21.1)	19 (100)
*A. dhakensis* (*n* = 18)	11 (61.1)	1 (5.6)	0 (0.0)	1 (5.6)	5 (27.8)	18 (100)
*A. hydrophila* (*n* = 10)	3 (30.0)	1 (10.0)	0 (0.0)	4 (40.0)	2 (20.0)	10 (100)
*A. media* (*n* = 2)	0 (0.0)	0 (0.0)	2 (100.0)	0 (0.0)	0 (0.0)	2 (100)
*A. trota* (*n* = 1)	1 (100.0)	0 (0.0)	0 (0.0)	0 (0.0)	0 (0.0)	1 (100)
*Total* (*n* = 74)	38 (51.4)	6 (8.1)	11 (14.9)	8 (10.8)	11 (14.9)	74 (100)

Percentages were calculated within each species; the Total row shows the distribution of all 74 isolates across the five isolation sources. The association between species and isolation source was statistically significant (Fisher’s exact test with Monte Carlo simulation, *p* < 0.001).

**Figure 2:**
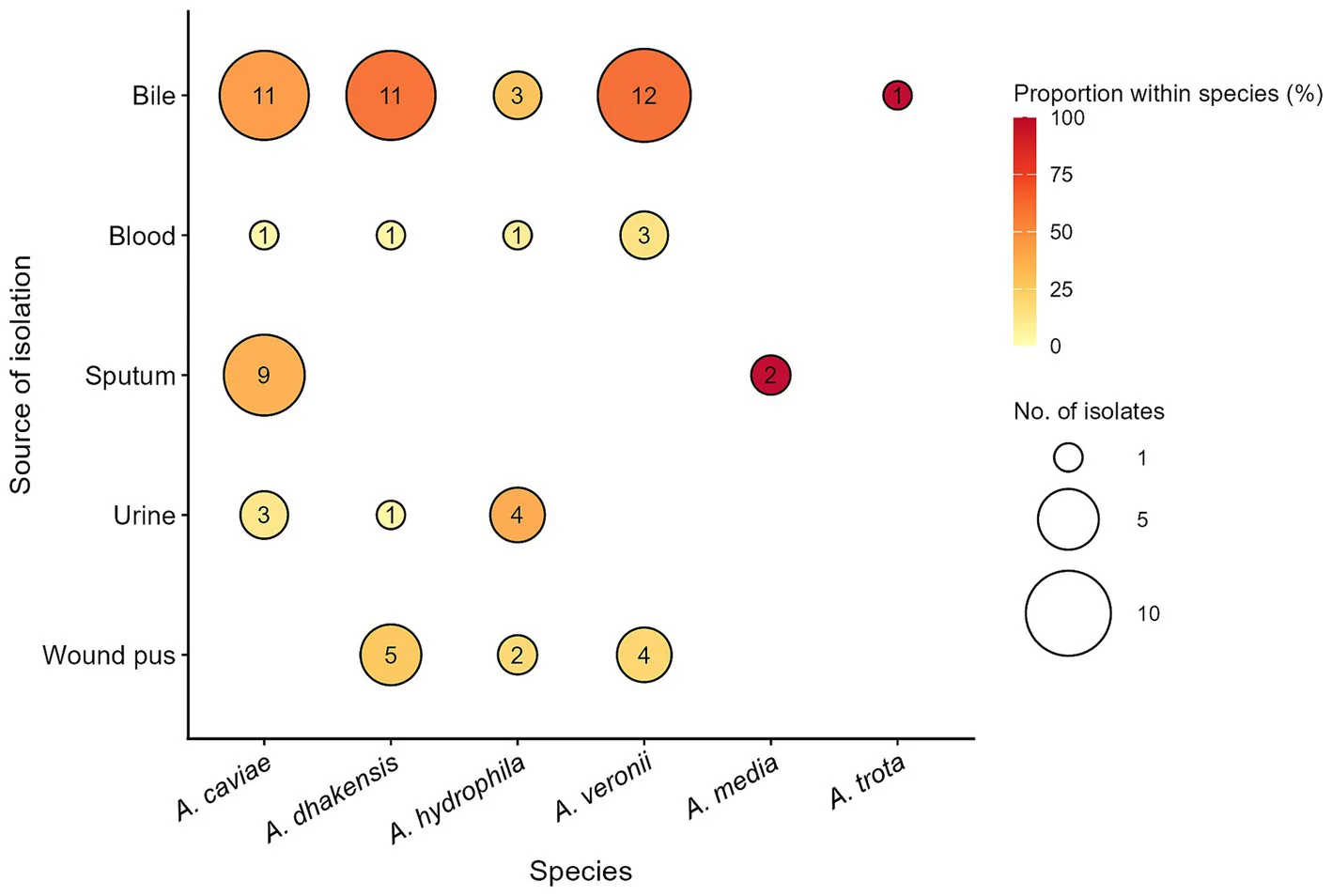
Bubble plot of the distribution of six *Aeromonas* species across five isolation sources (*n* = 74). The bubble area represents the absolute number of isolates; the bubble color represents the percentage of isolates from a given source relative to the total number of isolates of the same species; the number inside each bubble indicates the exact isolate count.

Fisher’s exact test with Monte Carlo simulation revealed a statistically significant association between species and source of isolation (*p* < 0.001; Pearson *χ*^2^ = 48.28, df = 20, reference *p* = 3.9 × 10^−4^). Analysis of standardized residuals identified three major contributors to this association: *A. media* in sputum (*r* = 3.12), *A. caviae* in sputum (*r* = 2.88) and *A. hydrophila* in urine (*r* = 2.81), all exceeding the significance threshold of |*r*| > 2, whereas *A. dhakensis* and *A. veronii* were under-represented in sputum (*r* = −1.64 and −1.68, respectively).

### Antimicrobial susceptibility profiles

The susceptibility of the 74 isolates to the 11 agents is summarized in Table 2. The highest resistance rate was observed for piperacillin/tazobactam (29.7%, 22/74), followed by trimethoprim/sulfamethoxazole (18.9%, 14/74), imipenem (16.2%, 12/74) and ceftazidime (16.2%, 12/74). Resistance to cefoperazone/sulbactam, meropenem, ciprofloxacin, cefepime, levofloxacin and aztreonam remained low (4.1–13.5%), and only one isolate (1.4%) was resistant to amikacin. No resistance to any of the 11 agents was detected in 39 isolates (52.7%). Resistance rates varied among the four major species (Supplementary Table S3): piperacillin/tazobactam resistance was more frequent in *A. veronii* (42.1%, 8/19) and *A. dhakensis* (38.9%, 7/18) than in *A. caviae* (16.7%, 4/24), whereas ciprofloxacin resistance was observed only in *A. caviae* (12.5%, 3/24), *A. veronii* (10.5%, 2/19) and *A. hydrophila* (10.0%, 1/10), with no resistant isolate among the *A. dhakensis*. Given the limited per-species sample sizes, these rates are descriptive only and were not formally compared.

**Table 2:** Antimicrobial susceptibility profiles of the 74 *Aeromonas* isolates against 11 antimicrobial agents.

Antimicrobial class	Agent	Susceptible, *n* (%)	Intermediate, *n* (%)	Resistant, *n* (%)
Penicillins	Piperacillin/tazobactam	52 (70.3)	0 (0.0)	22 (29.7)
Cephalosporins	Ceftazidime	62 (83.8)	0 (0.0)	12 (16.2)
	Cefoperazone/sulbactam	61 (82.4)	3 (4.1)	10 (13.5)
	Cefepime	66 (89.2)	3 (4.1)	5 (6.8)
Monobactams	Aztreonam	68 (91.9)	3 (4.1)	3 (4.1)
Carbapenems	Imipenem	59 (79.7)	3 (4.1)	12 (16.2)
	Meropenem	65 (87.8)	1 (1.4)	8 (10.8)
Fluoroquinolones	Ciprofloxacin	64 (86.5)	4 (5.4)	6 (8.1)
	Levofloxacin	61 (82.4)	9 (12.2)	4 (5.4)
Aminoglycosides	Amikacin	73 (98.6)	0 (0.0)	1 (1.4)
Sulfonamides	Trimethoprim/sulfamethoxazole	60 (81.1)	0 (0.0)	14 (18.9)

S, susceptible; I, intermediate; R, resistant. Results were interpreted according to CLSI guideline M45. Percentages were calculated with 74 isolates as the denominator.

### Distribution of antimicrobial resistance genes

A total of 491 non-redundant ARG records representing 85 distinct genes were predicted in the 74 genomes (Table 3 and Supplementary Data Sheet 1). Each isolate carried 2–24 ARGs (mean 6.6, median 5). According to the CARD Antibiotic Resistance Ontology annotations, the most frequently predicted resistance mechanism category was antibiotic inactivation—i.e., the presence of genes encoding hydrolytic or modifying enzymes such as β-lactamases, aminoglycoside-modifying enzymes and chloramphenicol acetyltransferases—accounting for 258 of the 491 records (52.5%), followed by antibiotic efflux (135, 27.5%), target alteration (65, 13.2%), target replacement (28, 5.7%) and target protection (5, 1.0%). These categories reflect the predicted function of the detected genes only; no expression or functional assays were performed. The RND efflux pump gene *rsmA* was the only gene detected in all isolates (100%). β-Lactamase genes were widespread across the collection: OXA-12-like penicillinases, the *cphA/imiH* metallo-β-lactamase family and MOX-type AmpC β-lactamases were detected in 63.5% (47/74), 58.1% (43/74) and 41.9% (31/74) of the isolates, respectively, and acquired β-lactamases including *bla_CTX-M-3_* (*n* = 2), *bla_PER-3_* (n = 3), *bla_VEB-1_* (*n* = 1) and the metallo-β-lactamase gene *bla_NDM-1_* (*n* = 3, 4.1%) were also identified. The three intrinsic β-lactamase families were strongly species-associated: OXA-12-like penicillinases were universal in *A. veronii*, *A. dhakensis* and *A. hydrophila* (19/19, 18/18 and 10/10, respectively) but absent from *A. caviae* (0/24); *cphA/imiH* genes showed a similar distribution (19/19, 14/18 and 10/10, respectively); and MOX-type AmpC genes were fixed in *A. caviae* (24/24) and detected only sporadically in *A. dhakensis* (4/18) and *A. hydrophila* (2/10), and in one of the two *A. media* isolates (1/2)—a species-marker-like distribution that independently corroborates the ANI-based species assignments (Bertran et al., 2021). Other frequently detected ARGs included the tetracycline resistance gene *tet(E)* (41.9%), the class 1 integron-associated genes *sul1* and *qacEΔ1* (23.0% each, co-detected in 16 of the 17 *sul1*-positive isolates), macrolide phosphotransferase genes (*mphA*/*mrx*), dihydrofolate reductase genes (*dfrA12/14/15*), quinolone resistance genes (*qnrS2*, *qnrVC1*), aminoglycoside-modifying enzyme genes (23.0%) and *mcr-3* variants (8.1%) (Table 3 and Supplementary Table S2).

**Table 3:** Antimicrobial resistance genes detected in three or more of the 74 *Aeromonas* isolates (*n* = 41 genes), ranked by detection rate in descending order.

Resistance gene	Drug class(es) (CARD)	Gene family	Isolates, *n*	Detection rate, %
*rsmA*	Fluoroquinolone antibiotic; diaminopyrimidine antibiotic; phenicol antibiotic	Resistance-nodulation-cell division (RND) antibiotic efflux pump	74	100.0
*EF-Tu*	Elfamycin antibiotic	Elfamycin resistant EF-Tu	59	79.7
*tet(E)*	Tetracycline antibiotic	Major facilitator superfamily (MFS) antibiotic efflux pump	31	41.9
*imiH*	Carbapenem	CphA beta-lactamase	18	24.3
*cphA3*	Carbapenem	CphA beta-lactamase	18	24.3
*sul1*	sulfonamide antibiotic	Sulfonamide resistant sul	17	23.0
*qacEΔ1*	Disinfecting agents and antiseptics	Major facilitator superfamily (MFS) antibiotic efflux pump	17	23.0
*OXA-912*	Penicillin beta-lactam	OXA beta-lactamase; OXA-12-like beta-lactamase	15	20.3
*OXA-951*	Penicillin beta-lactam	OXA beta-lactamase; OXA-12-like beta-lactamase	14	18.9
*mphA*	Macrolide antibiotic	Macrolide phosphotransferase (MPH)	13	17.6
*mrx*	Macrolide antibiotic	Macrolide phosphotransferase (MPH)	13	17.6
*AQU-2*	Cephalosporin	AQU beta-lactamase	11	14.9
*aadA2*	Aminoglycoside antibiotic	ANT(3″)	8	10.8
*dfrA12*	Diaminopyrimidine antibiotic	Trimethoprim resistant dihydrofolate reductase dfr	8	10.8
*aadA*	Aminoglycoside antibiotic	ANT(3″)	7	9.5
*OXA-1161*	Penicillin beta-lactam	OXA beta-lactamase	7	9.5
*OXA-950*	Penicillin beta-lactam	OXA beta-lactamase; OXA-12-like beta-lactamase	7	9.5
*MOX-20*	Cephalosporin; penicillin beta-lactam	MOX beta-lactamase	7	9.5
*APH(3′)-Ia*	Aminoglycoside antibiotic	APH(3′)	6	8.1
*cphA2*	Carbapenem	CphA beta-lactamase	6	8.1
*MOX-25*	Cephalosporin; penicillin beta-lactam	MOX beta-lactamase	6	8.1
*OXA-1163*	Penicillin beta-lactam	OXA beta-lactamase	6	8.1
*OXA-724*	Penicillin beta-lactam	OXA beta-lactamase; OXA-12-like beta-lactamase	5	6.8
*MOX-15*	Cephalosporin; penicillin beta-lactam	MOX beta-lactamase	5	6.8
*catB3*	Phenicol antibiotic	chloramphenicol acetyltransferase (CAT)	4	5.4
*OXA-780*	Penicillin beta-lactam	OXA beta-lactamase	4	5.4
*floR*	Phenicol antibiotic	Major facilitator superfamily (MFS) antibiotic efflux pump	4	5.4
*qnrS2*	Fluoroquinolone antibiotic	Quinolone resistance protein (qnr)	4	5.4
*tet(A)*	Tetracycline antibiotic	Major facilitator superfamily (MFS) antibiotic efflux pump	3	4.1
*tet(C)*	Tetracycline antibiotic	Major facilitator superfamily (MFS) antibiotic efflux pump	3	4.1
*TEM-1*	Monobactam; cephalosporin; penicillin beta-lactam	TEM beta-lactamase	3	4.1
*PER-3*	Monobactam; carbapenem; cephalosporin; penicillin beta-lactam	PER beta-lactamase	3	4.1
*OXA-1041*	Penicillin beta-lactam	OXA beta-lactamase	3	4.1
*MOX-8*	Cephalosporin; penicillin beta-lactam	MOX beta-lactamase	3	4.1
*MOX-3*	Cephalosporin; penicillin beta-lactam	MOX beta-lactamase	3	4.1
*NDM-1*	Carbapenem; cephalosporin; penicillin beta-lactam	NDM beta-lactamase	3	4.1
*mcr-3.32*	Peptide antibiotic	MCR phosphoethanolamine transferase	3	4.1
*AAC(6′)-Ib10*	Aminoglycoside antibiotic	AAC(6′)	3	4.1
*APH(6)-Id*	Aminoglycoside antibiotic	APH(6)	3	4.1
*catII*	Phenicol antibiotic	chloramphenicol acetyltransferase (CAT)	3	4.1
*MOX-19*	Cephalosporin; penicillin beta-lactam	MOX beta-lactamase	3	4.1

Genes are ranked by detection rate. The remaining 44 genes, each detected in one or two isolates, are listed in Supplementary Table S2. ARG prediction: CARD hits with amino-acid identity ≥ 80% and query coverage ≥ 90%; duplicated hits per isolate were collapsed. Drug classes and gene families follow the CARD Antibiotic Resistance Ontology (ARO). MFS, major facilitator superfamily; RND, resistance-nodulation-cell division; MPH, macrolide phosphotransferase; CAT, chloramphenicol acetyltransferase. BRP(MBL), a bleomycin-resistance protein, is annotated under glycopeptide antibiotics in CARD because bleomycin is itself a glycopeptide. β-lactamase genes are listed without the *bla* prefix.

### Genetic context of *bla_NDM-1_*

In the two imipenem- and meropenem-resistant isolates A12527 (*A. hydrophila*) and A14504 (*A. caviae*), *bla_NDM-1_* was carried on a 6,558-bp contig that was 100% identical between the two strains and comprised a truncated Tn125-like module: an internal ISAba125 fragment, a unique 753-bp region, the ISAba125 3′ end harboring the −35 promoter box, *bla_NDM-1_*, *bleMBL*, *trpF* and *tat*, flanked on both sides by uncharacterized multi-copy repeats that terminated the assemblies. No known plasmid replicon was detected on the contig (PlasmidFinder database), and the contig GC content (56.8%) was lower than that of the corresponding assemblies (61.0%). Given that the chromosomal *groEL* sequences of the two strains share only 92.1% nucleotide identity, the complete identity of this 6,558-bp element across the species boundary indicates recent horizontal transfer of a mobile element, most likely plasmid-borne, between *A. hydrophila* and *A. caviae* in our setting (Figure 3). In the meropenem-resistant but imipenem-susceptible isolate A14509, *bla_NDM-1_* was embedded in a distinct, decayed context on a 2,133-bp contig: the ISAba125 3′ end and *bla_NDM-1_* were intact, but *bleMBL* was truncated (~190 of 366 bp), the remaining downstream module (*trpF*, *tat*, *groES*, *groEL*) was absent, and the flanking regions were unrelated to those of the other two isolates and associated with ISCR-family-like elements. Notably, the 197 bp immediately upstream of the *bla_NDM-1_* start codon, including the ISAba125-derived −35 box (TTGAAA) and the complete intergenic spacer, were 100% identical in all three isolates, indicating that the imipenem susceptibility of A14509 cannot be attributed to loss of the proximal promoter sequence (Figure 3).

**Figure 3:**
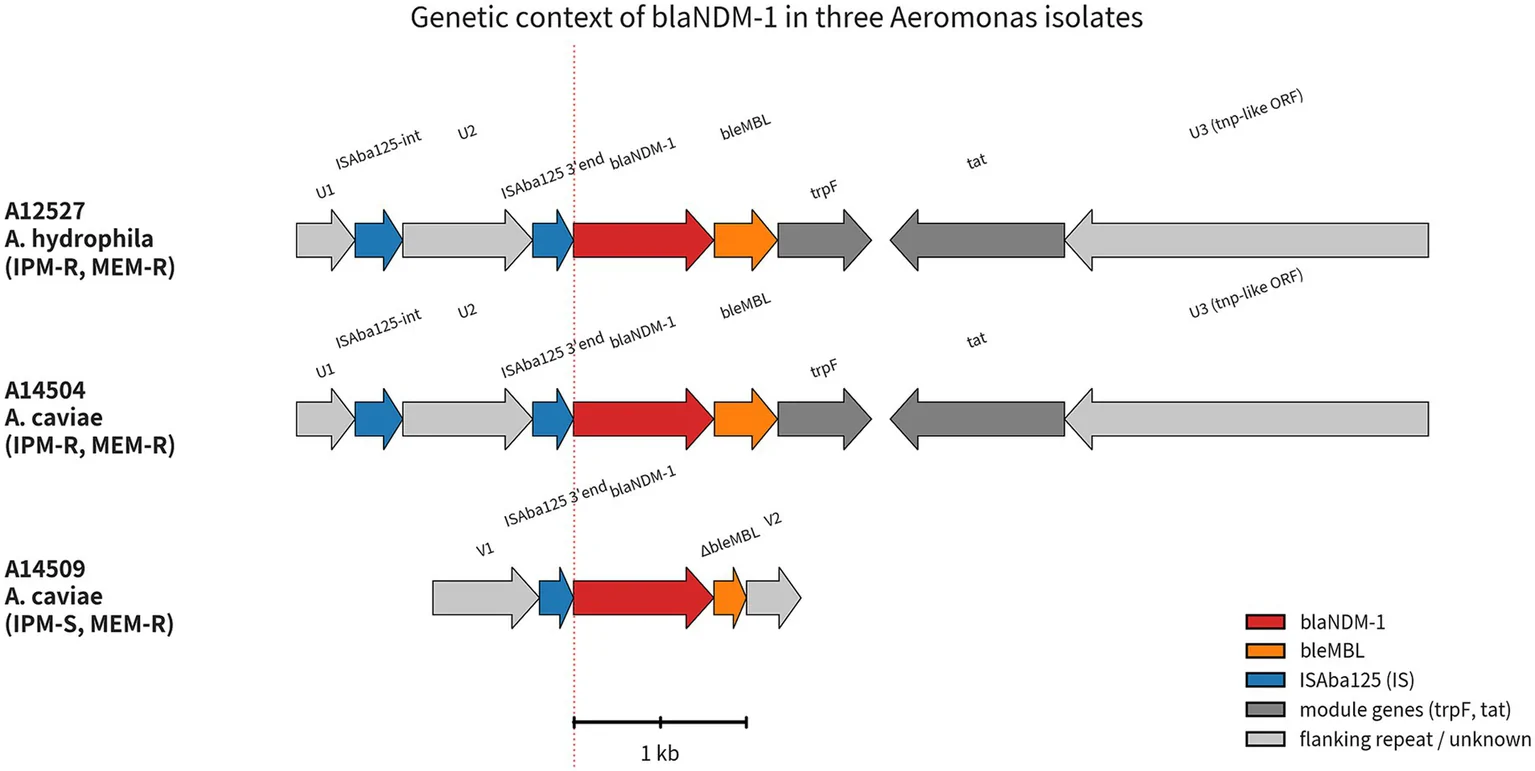
Genetic context of *bla_NDM-1_* in the three *bla_NDM-1_*-positive isolates. Tracks are aligned at the *bla_NDM-1_* start codon; genes are shown as arrows (*bla_NDM-1_*, red; *bleMBL*, orange; ISAba125 remnants, blue; other module genes, dark gray; uncharacterized repeats and flanking regions, light gray). The ISAba125-derived −35 box (TTGAAA) and the intergenic spacer are 100% identical in all three isolates. IPM, imipenem; MEM, meropenem; R, resistant; S, susceptible.

### Correlation between resistance genotypes and phenotypes

Agreement between the predicted genotypes and the observed phenotypes varied markedly among drug classes (Table 4). For sulfonamides, carriage of *sul1* and/or *dfrA* genes showed substantial agreement with the SXT-resistant phenotype (*κ* = 0.773, Fisher’s exact *p* < 0.0001), with a sensitivity of 100% (14/14) and a specificity of 90.0% (54/60); dfrA alone achieved almost perfect agreement (*κ* = 0.856) without any false-positive call (Table 5). For fluoroquinolones, *qnr* carriage agreed substantially with ciprofloxacin/levofloxacin resistance (*κ* = 0.706, *p* < 0.0001). Statistically significant but only fair agreement was observed for carbapenems (*κ* = 0.218, *p* = 0.0028), cephalosporins (*κ* = 0.175, *p* = 0.0067) and monobactams (*κ* = 0.364, *p* = 0.0222): although every carbapenem- or cephalosporin-resistant isolate carried the corresponding β-lactamase genes (sensitivity 100%), the same genes were also widespread among susceptible isolates, resulting in low specificity. Carriage of the *cphA/imiH* family showed only a borderline association with carbapenem resistance (*p* = 0.060) and a low positive predictive value (25.6%, 11/43), whereas *bla_NDM-1_* was detected in 3 of the 13 carbapenem-resistant isolates and in no susceptible isolate (*p* = 0.0044) (Table 5). No association could be established for piperacillin/tazobactam, because all 74 isolates carried at least one β-lactamase gene, nor for amikacin, for which 16 of the 17 carriers of aminoglycoside resistance genes remained phenotypically susceptible (*p* = 0.2297).

**Table 4:** Concordance between resistance genotypes and phenotypes at the antimicrobial-class level (*n* = 74 isolates).

Antimicrobial class (agents)	Genotype+, *n*	Phenotype R, *n*	TP/FP/FN/TN	Sensitivity, %	Specificity, %	Agreement, %	Cohen’s *κ*	Fisher’s exact *P*
Penicillins (TZP)	74	22	22/52/0/0	100.0	0.0	29.7	0.000	1.0000
Cephalosporins (CAZ, SCF, FEP)	51	13	13/38/0/23	100.0	37.7	48.6	0.175	0.0067
Monobactams (ATM)	7	3	2/5/1/66	66.7	93.0	91.9	0.364	0.0222
Carbapenems (IPM, MEM)	47	13	13/34/0/27	100.0	44.3	54.1	0.218	0.0028
Fluoroquinolones (CIP, LEV)	5	6	4/1/2/67	66.7	98.5	95.9	0.706	<0.0001
Aminoglycosides (AMK)	17	1	1/16/0/57	100.0	78.1	78.4	0.088	0.2297
Sulfonamides (SXT)	20	14	14/6/0/54	100.0	90.0	91.9	0.773	<0.0001

Genotype+, carriage of at least one CARD-annotated ARG of the corresponding drug class (the ubiquitous *rsmA* efflux pump gene excluded); Phenotype R, resistance to any agent of the class; TP, true positive; FP, false positive; FN, false negative; TN, true negative. Sensitivity/specificity/agreement were calculated with the phenotype as the reference. Fisher’s exact test, two-sided. TZP, piperacillin/tazobactam; CAZ, ceftazidime; SCF, cefoperazone/sulbactam; FEP, cefepime; ATM, aztreonam; IPM, imipenem; MEM, meropenem; CIP, ciprofloxacin; LEV, levofloxacin; AMK, amikacin; SXT, trimethoprim/sulfamethoxazole. For the contingency-table and Cohen’s *κ* analyses, resistant (R) isolates were scored as phenotype-positive and intermediate (I) isolates were grouped with susceptible (S) isolates.

**Table 5:** Association between key resistance genes and the corresponding resistance phenotypes (*n* = 74 isolates).

Gene/gene family	Comparator phenotype	TP/FP/FN/TN	Sensitivity, %	PPV, %	Cohen’s *κ*	Fisher’s exact *P*
*cphA/imiH* family (metallo-beta-lactamase)	Carbapenems	11/32/2/29	84.6	25.6	0.169	0.0602
*bla_NDM-1_*	Carbapenems	3/0/10/61	23.1	100.0	0.331	0.0044
*MOX-type* AmpC beta-lactamases	Ceftazidime	6/25/6/37	50.0	19.4	0.059	0.5426
*OXA-12-like* beta-lactamases	Piperacillin/tazobactam	18/29/4/23	81.8	38.3	0.196	0.0382
*sul1*	Trimethoprim/sulfamethoxazole	11/6/3/54	78.6	64.7	0.634	<0.0001
*dfrA* (*dfrA12*/14/15)	Trimethoprim/sulfamethoxazole	11/0/3/60	78.6	100.0	0.856	<0.0001
*sul1* or *dfrA*	Trimethoprim/sulfamethoxazole	14/6/0/54	100.0	70.0	0.773	<0.0001
*qnr* (*qnrS2*/*qnrVC1*)	Ciprofloxacin/levofloxacin	4/1/2/67	66.7	80.0	0.706	<0.0001

PPV, positive predictive value. The *cphA/imiH* family comprises *cphA2/cphA3/cphA7/imiH*; *MOX-type* genes comprise nine *MOX* variants; *OXA-12-like* genes comprise eight *OXA-12-like* variants. Fisher’s exact test, two-sided. For the contingency-table and Cohen’s κ analyses, resistant (R) isolates were scored as phenotype-positive and intermediate (I) isolates were grouped with susceptible (S) isolates.

### Putative virulence factor profiles and their distribution by species and isolation source

A total of 56 VFs were detected across the 74 isolates, with each isolate carrying 28–40 VFs (median 31, IQR 30–36). Twenty-six VFs were present in all isolates (100% carriage; including T2SS, T4SS, flagella, type IV pili, LPS/LOS/O-antigen, capsule, the AcrAB efflux pump and catalase), constituting a conserved core virulome; 10 VFs were each found in only a single isolate (carriage < 2%). The remaining 20 variable VFs (carriage 2.7–98.6%) were retained for downstream analyses (Figure 4).

**Figure 4:**
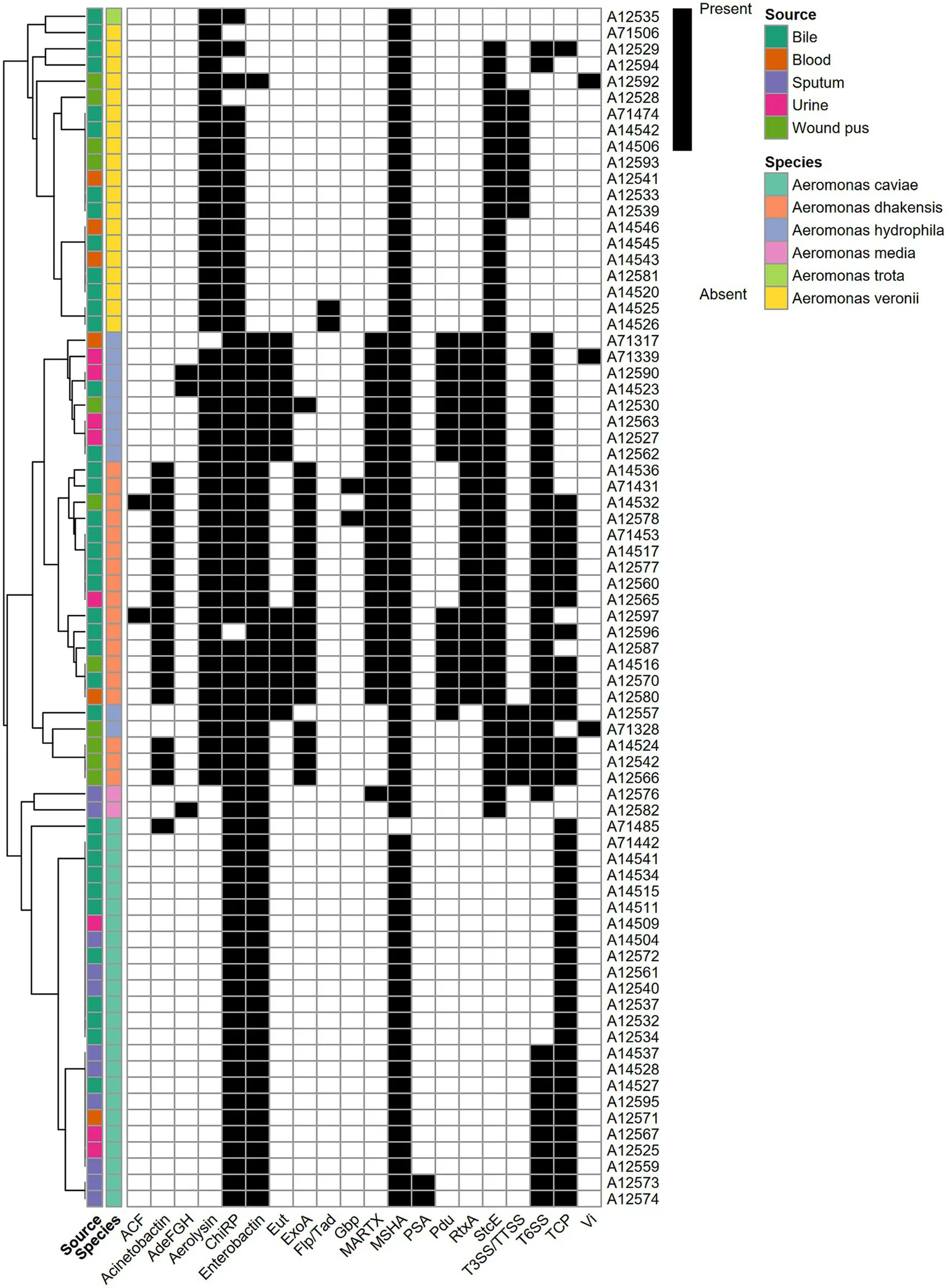
Presence/absence heatmap of the 20 variable putative virulence factors (carriage 2–99%) across the 74 isolates. Rows represent isolates (clustered by UPGMA on Jaccard distances) and columns represent VFs; black cells indicate carriage and white cells absence. Annotation bars indicate species and isolation source.

The VF load differed significantly among species (Kruskal–Wallis *χ*^2^ = 56.15, df = 5, *p* < 0.001): *A. dhakensis* carried the most VFs (median 37, IQR 37–38), followed by *A. hydrophila* (36, IQR 36–37), both significantly higher than *A. caviae* (30, IQR 30–31; adjusted *p* = 3.5 × 10^−7^ and 1.4 × 10^−5^, respectively) and *A. veronii* (31, IQR 30–31; adjusted *p* = 1.2 × 10^−6^ and 3.6 × 10^−5^, respectively). *A. hydrophila* also differed from *A. dhakensis* (adjusted *p* = 0.023), whereas *A. caviae* and *A. veronii* did not (adjusted *p* = 0.682). VF load did not differ among isolation sources (*χ*^2^ = 5.10, df = 4, *p* = 0.277).

Of the 20 variable VFs, 13 showed significantly different carriage rates among species (Fisher’s exact test, BH-adjusted *p* < 0.05; Figure 4), 11 of them at adjusted *p* < 0.001. The key effectors were strongly species-structured: aerolysin was ubiquitous in *A. dhakensis*, *A. veronii* and *A. hydrophila* (90–100%) but, like ExoA and the StcE protease, entirely absent from *A. caviae*; ExoA, the MARTX/RtxA cytotoxins and T6SS predominated in *A. dhakensis* and *A. hydrophila*, the acinetobactin siderophore was almost exclusive to *A. dhakensis*, and the Eut/Pdu metabolic islands were most frequent in *A. hydrophila*; TCP pili predominated in *A. caviae* and *A. dhakensis*, and enterobactin was carried by all major species except *A. veronii*. T3SS/TTSS (most frequent in *A. veronii*) and the AdeFGH efflux pump also differed significantly among species (both adjusted *p* = 0.010), whereas MSHA, ChiRP, PSA, Flp/Tad, Gbp, ACF and Vi did not (Figure 4 and Supplementary Data Sheet 2).

By isolation source, three variable VFs differed significantly: aerolysin (adjusted *p = 0.002; w*ound pus 100%, bile 71.1%, blood 66.7%, urine 62.5%, sputum 0%), StcE (adjusted *p* = 0.006; wound pus 100% vs. sputum 18.2%) and T3SS/TTSS (adjusted *p* = 0.006; wound pus 63.6% vs. 0% in sputum and urine).

Principal coordinates analysis (Figure 5) showed that the first two axes explained 40.3 and 31.5% of the variation in VF profiles, respectively, with isolates clustering distinctly by species. PERMANOVA confirmed species as the dominant structuring factor [*R*^2^ = 0.797, *F*(5,68) = 53.35, *p* = 0.001]; within-group dispersions were homogeneous (betadisper *p* = 0.375), indicating genuine differences in group centroids, and all pairwise comparisons among the four major species were significant (adjusted *p* = 0.001 for all; *R*^2^ = 0.437–0.795). Isolation source had a significant but weaker effect [*R*^2^ = 0.169, *F*(4,69) = 3.52, *p* = 0.002]; however, dispersions were heterogeneous across sources (betadisper *p* = 0.001), warranting cautious interpretation. Importantly, because isolation source and species composition were strongly coupled in this cohort (e.g., sputum isolates were almost exclusively *A. caviae* and *A. media*, whereas wound-pus isolates were dominated by *A. dhakensis*, *A. veronii* and *A. hydrophila*), the source-related PERMANOVA signal is partly confounded by species identity and should not be over-interpreted as a genuine source effect (see Discussion). Pairwise comparisons showed significant differences for sputum vs. wound pus (*R*^2^ = 0.440, adjusted *p* = 0.005), sputum vs. bile (*R*^2^ = 0.125, adjusted *p* = 0.005), sputum vs. blood (*R*^2^ = 0.312, adjusted *p* = 0.013), sputum vs. urine (*R*^2^ = 0.265, adjusted *p* = 0.022) and wound pus vs. urine (*R*^2^ = 0.193, adjusted *p* = 0.013); all other pairs were non-significant.

**Figure 5:**
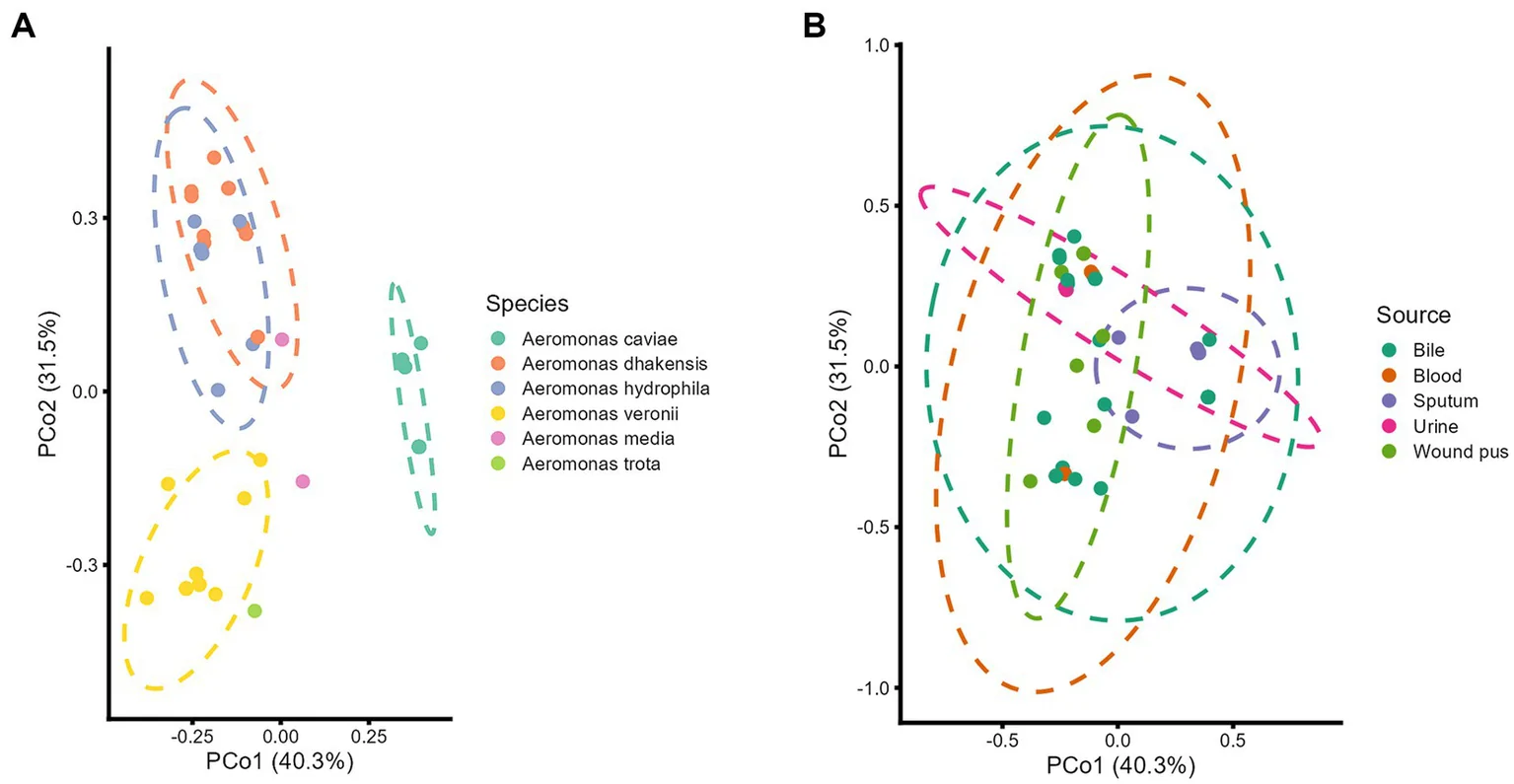
Principal coordinates analysis (PCoA) of Jaccard distances based on the variable-VF presence/absence matrix. The first two axes explain 40.3 and 31.5% of the variation, respectively. Points represent isolates colored **(A)** by species and **(B)** by isolation source; dashed ellipses indicate 95% confidence regions for groups with ≥ 3 isolates.

## Discussion

### Clinical ecology and species–site associations

In this cohort of 74 clinical *Aeromonas* isolates from extra-intestinal clinical specimens, the species spectrum was dominated by *A. caviae*, *A. veronii* and *A. dhakensis*, which together accounted for 82.4% of all isolates. This pattern is broadly consistent with contemporary clinical series in which *A. caviae*, *A. hydrophila* and *A. veronii* have traditionally predominated (Janda and Abbott, 2010; Parker and Shaw, 2011; Fernández-Bravo and Figueras, 2020), and with a recent 14-year retrospective study from the United States (Pineda-Reyes et al., 2024), a multicenter prospective cohort from Japan (Sakurai et al., 2023), and Chinese series of intestinal and extra-intestinal isolates (Zhou et al., 2019; Lu et al., 2025). Notably, *A. dhakensis* represented nearly one-quarter of our isolates (24.3%) and ranked as the second most common species, exceeding *A. hydrophila* (13.5%). Because *A. dhakensis* is frequently misidentified as *A. hydrophila* by conventional phenotypic methods, its clinical significance has historically been underestimated; genome-based identification, as used here, has revealed *A. dhakensis* to be more prevalent and more virulent than previously recognized (Chen et al., 2014a; Chen et al., 2016). This pattern was reproduced in our own cohort: MALDI-TOF MS failed to identify any of the 18 *A. dhakensis* isolates at the species level—half of them being reported as *A. hydrophila*—and achieved species-level concordance with ANI assignments in only 44.6% of the entire collection, underscoring that genome-based identification is currently indispensable for accurate species-level epidemiology of clinical aeromonads (Vieira et al., 2025). The complete failure to identify *A. dhakensis* most likely reflects its absence from the VITEK MS IVD knowledge base (KB V3.2) in use during the study period, as MALDI-TOF MS with updated databases can correctly identify this species (Chen et al., 2014b; Kitagawa et al., 2022; Verma et al., 2026). Our core-genome phylogeny, in which all 74 isolates formed six clades fully concordant with ANI assignments, further confirms the robustness of genome-based species delineation and illustrates the extreme genetic diversity of the collection (71 STs, 39 of them novel).

More than half of the isolates (51.4%) were recovered from bile, making the biliary tract the principal reservoir of clinical *Aeromonas* in our setting. This agrees with reports that the hepatobiliary system is an important focus of *Aeromonas* infection, particularly in patients with cholelithiasis, hepatobiliary malignancy or a history of biliary instrumentation, in whom ascending translocation from the intestinal tract is facilitated (Chan et al., 2000; Chao et al., 2013; Henrichs et al., 2026). The cited biliary series (Chan et al., 2000; Chao et al., 2013) attribute biliary *Aeromonas* infection mainly to these predisposing conditions rather than to food-associated acquisition; although an intestinal reservoir seeded by contaminated food or water is plausible for these food- and waterborne organisms, whether the biliary isolates in our cohort originated from gastroenteritis-related intestinal carriage could not be assessed, as dietary histories and stool cultures were not collected. The demographic profile—predominantly male (68.9%) and elderly (median age 69 years; 75.7% ≥ 60 years)—also mirrors the recognized predilection of invasive *Aeromonas* infection for older patients with underlying hepatobiliary or other chronic diseases (Janda and Abbott, 2010; Chen et al., 2016). Beyond the overall predominance of bile isolates, the significant non-random association between species and isolation site (*p* < 0.001) is of particular interest: *A. caviae* was enriched in sputum and accounted for 81.8% (9/11) of respiratory isolates, *A. hydrophila* was enriched in urine, *A. dhakensis* predominated in wound pus, and *A. veronii* was the species most frequently isolated from blood (3/6). The wound-pus predominance of *A. dhakensis* accords with its recognized role in skin and soft-tissue infection and its greater cytotoxicity (Chen et al., 2014a; Chen et al., 2016), and the bloodstream recovery of *A. veronii* is consistent with its established role in bacteremia (Chen et al., 2016). These site-specific patterns may reflect genuine differences in tissue tropism and VF repertoires among species, although the small numbers in some strata preclude firm conclusions for the rarer species.

### Antimicrobial resistance phenotypes and the limits of resistome prediction

Overall, the isolates exhibited low-to-moderate resistance to most agents tested, with piperacillin/tazobactam standing out at 29.7%, whereas amikacin retained nearly full activity. This pattern is consistent with previous surveillance of clinical and environmental *Aeromonas*, in which reduced susceptibility to penicillins and early cephalosporins is common but fluoroquinolone and aminoglycoside resistance remains sporadic (Pineda-Reyes et al., 2024). The high frequency of piperacillin/tazobactam resistance is mechanistically plausible: aeromonads constitutively or inducibly produce up to three β-lactamases—a penicillinase (OXA-12-like), an inducible cephalosporinase (AmpC/MOX-type) and a metallo-β-lactamase (CphA/ImiH)—whose coordinated expression confers resistance to most β-lactams except fourth-generation cephalosporins and, usually, carbapenems (Rossolini et al., 1996). In line with this model, *OXA-12-like* genes were the most prevalent β-lactamase genes in our collection (63.5%).

A central finding of this study is the striking genotype–phenotype discrepancy for β-lactams. Every isolate carried at least one β-lactamase gene, and the *cphA/imiH* family—encoding a chromosome-borne metallo-β-lactamase capable of hydrolysing carbapenems—was found in 58.1% of the isolates, yet only 16.2 and 10.8% were resistant to imipenem and meropenem, respectively. *cphA/imiH* carriage showed only a borderline association with carbapenem resistance (*p* = 0.060), and its positive predictive value was only 25.6%. This mirrors the classical description of *Aeromonas* β-lactamases as tightly regulated, inducible enzymes that are phenotypically silent in most wild-type cells but can be derepressed by mutation or induction during β-lactam exposure (Rossolini et al., 1996; Walsh et al., 1997; Wu et al., 2012). Recent large-scale surveillance supports this interpretation at the population level: in the multicentre European AEuROMONAS collection of 590 bloodstream *Aeromonas* isolates (2020–2024), susceptibility to aztreonam, ceftazidime and cefepime exceeded 90% at the genus level despite the chromosomally encoded β-lactamase armamentarium of the genus, and resistance rates differed among species, underscoring that regulated expression—rather than gene presence alone—determines the resistance phenotype in clinical isolates (Boattini et al., 2026). Consequently, resistome-based prediction alone would substantially overestimate carbapenem resistance in this genus, and genotypic screening should be interpreted together with phenotypic confirmation before guiding therapy. Conversely, the high prevalence of the *cphA/imiH* family in this collection constitutes a considerable hidden reservoir of inducible carbapenemase activity that may be selected during carbapenem treatment and should be taken into account in antimicrobial stewardship. Consistent with this notion, high rates of carbapenem resistance have been reported among clinical *A. dhakensis* isolates in Southeast Asia (Puah et al., 2022; Chen et al., 2025).

Of particular concern is the detection of *bla_NDM-1_* in three isolates (4.1%). NDM enzymes hydrolyze virtually all β-lactams including carbapenems, and their emergence in *Aeromonas*—a genus closely associated with aquatic environments—underscores the role of waterborne bacteria as reservoirs and vectors of clinically critical resistance determinants (Singh et al., 2026); *bla_NDM-1_*-positive *A. caviae* has already been reported from human infection in China (Luo et al., 2022, 2023; Xu et al., 2022; Hu et al., 2023). Notably, the two imipenem- and meropenem-resistant isolates—a urine-derived *A. hydrophila* (A12527) and a sputum-derived *A. caviae* (A14504)—carried a 6,558-bp *bla_NDM-1_* element that was 100% identical across the species boundary, pointing to recent horizontal transfer of the element within our setting (Figure 3). This inference is based on comparative sequence analysis rather than on transfer experiments: the two strains are not clonally related (chromosomal *groEL* identity 92.1%), yet share the 6,558-bp element, including non-coding flanks, at 100% identity, and the element carries Tn125/ISAba125-derived mobilization signatures with a GC content (56.8%) deviating from the host assemblies (61.0%). Spontaneous, independent acquisition of an identical element by two distantly related strains is highly unlikely; recent horizontal transfer of a mobile, most likely plasmid-borne, element within the shared hospital environment is the parsimonious explanation, although acquisition from an unsampled common source cannot be excluded. No conjugation or mating assays were performed in this retrospective study. As in previous reports of non-plasmid-encoded *bla_NDM-1_* in *A. caviae* (Xu et al., 2022; Hu et al., 2023), no plasmid backbone could be resolved, because our analysis relied on short-read (Illumina) draft assemblies: the *bla_NDM-1_*-carrying contigs were truncated by multi-copy repetitive elements in all three isolates, and whether the element is plasmid-borne or chromosomally integrated could not be resolved; long-read sequencing (e.g., Oxford Nanopore) of these isolates would be required to establish the complete genetic environment and location of *bla_NDM-1_*. The carriage of an additional, highly active carbapenemase in these two isolates translated more directly into phenotype than the indigenous *cphA/imiH* background; the third isolate (A14509) was resistant to meropenem but remained susceptible to imipenem although its *bla_NDM-1_* gene and the ISAba125-derived −35 promoter box were intact at the sequence level (see Results, ‘Genetic context of blaNDM-1’), pointing to substrate-dependent differences in expression or activity rather than gene disruption; quantitative expression assays will be required to resolve this discrepancy.

In contrast to β-lactams, the genotype–phenotype agreement for trimethoprim/sulfamethoxazole was substantial to almost perfect (κ up to 0.856), with a combined sensitivity of 100% for *sul1*/*dfrA* carriage. The tight coupling of *sul1* with *qacEΔ1* in 16 of 17 isolates strongly suggests that these genes reside on class 1 integron-borne gene cassettes, which are frequently mobilized on plasmids and may disseminate among *Enterobacterales* and aeromonads in shared niches (Partridge et al., 2009). The excellent concordance indicates that WGS-based resistome analysis can reliably predict the SXT phenotype in *Aeromonas*. A similarly strong, albeit less sensitive, association was observed for fluoroquinolones: all five *qnr*-positive isolates but one were non-susceptible to ciprofloxacin or levofloxacin, whereas two resistant isolates lacked any known acquired quinolone resistance gene.

The lack of association for aminoglycosides illustrates another caveat of resistome interpretation: 17 isolates carried aminoglycoside-modifying enzyme genes (predominantly *aadA*, which confers streptomycin/spectinomycin rather than amikacin resistance), but only one isolate was phenotypically resistant to amikacin. Drug-class-level mapping in CARD overestimates phenotype when the annotated substrate spectrum of a gene does not cover the tested agent, emphasizing the need to match genes to agent-specific phenotypes wherever substrate information is available. Likewise, the universal carriage of the RND efflux pump gene *rsmA* confirms that efflux-mediated intrinsic resistance is part of the core resistome of *Aeromonas* rather than an acquired trait. The detection of *mcr-3* variants in six isolates (8.1%) further adds to the growing evidence that *Aeromonas* spp. constitute a natural reservoir of MCR-3-family colistin resistance determinants (Yu et al., 2023; Guo et al., 2024), including *mcr-3*-carrying isolates recovered from coastal waters of Zhejiang Province, China (Chen et al., 2024); although colistin was not included in our susceptibility panel, the presence of this reservoir warrants continued surveillance given the last-line role of polymyxins.

### Species-stratified virulence potential

The virulome analysis adds a further dimension to the species-level stratification observed for clinical distribution and resistance. Twenty-six VFs—including T2SS, T4SS, flagella, type IV pili, LPS/LOS/O-antigen, capsule and the AcrAB efflux pump—were present in every isolate, forming a conserved genus-level core virulome that confers a baseline capacity for colonization, motility, adhesion and host interaction (Janda and Abbott, 2010; Tomás, 2012); it is the accessory (variable) VFs that discriminate among isolates. Species was the dominant driver of VF-profile differentiation, explaining approximately 80% of the variation (PERMANOVA *R*^2^ = 0.797), a result not confounded by differences in within-group dispersion, and the four major species were all pairwise distinguishable. This strong species specificity of the predicted virulence determinants is consistent with population-genomic studies of the genus (Zhong et al., 2019; Abdella et al., 2023; Singh et al., 2026).

*Aeromonas dhakensis* and *A. hydrophila* carried the highest VF loads (medians 37 and 36), significantly exceeding *A. caviae* and *A. veronii*, and were highly enriched for key effectors including aerolysin, ExoA, MARTX, RtxA and T6SS. Aerolysin, the major pore-forming toxin of aeromonads, is associated with the severity of gastroenteritis and sepsis (Tomás, 2012); in our cohort it was carried by 100% of *A. dhakensis* and *A. veronii* but was completely absent from *A. caviae*, mirroring the species-specific distribution reported previously (Tomás, 2012; Zhong et al., 2019). ExoA, an ADP-ribosylating toxin, and T3SS, both key determinants of acute infection, were likewise restricted mainly to *A. dhakensis*, *A. hydrophila* and *A. veronii*, further supporting the greater potential virulence of these species—*A. dhakensis* in particular, whose virulence advantage has been repeatedly documented in severe infections such as necrotizing fasciitis and sepsis (Chen et al., 2014a; Chen et al., 2016). Comparative pathogenomic analysis of the *A. hydrophila* virulome has likewise resolved a broad spectrum of conserved and accessory virulence determinants, supporting the effector enrichment observed in this species (Abdella et al., 2023). The enrichment of the MARTX/RtxA cytotoxins and the acinetobactin iron-uptake system in *A. dhakensis*, and of the Eut/Pdu metabolic virulence islands in *A. hydrophila*, suggests that different species employ distinct virulence strategies to adapt to the host (Zhong et al., 2019). In contrast, *A. caviae* lacked aerolysin, ExoA and StcE and had the lowest VF load, consistent with its comparatively low clinical virulence (Janda and Abbott, 2010), whereas the universal carriage of TCP pili in this species may represent a species-specific adhesion and colonization strategy.

The effect of isolation source on the virulome was weaker (*R*^2^ = 0.169) and should be interpreted with caution. Within-group dispersions were heterogeneous across sources (betadisper *p* = 0.001), which can inflate PERMANOVA significance; moreover, source and species composition were strongly coupled in this cohort—sputum isolates were almost exclusively *A. caviae* and *A. media* (9/11 and 2/11, respectively), whereas wound-pus isolates were dominated by *A. dhakensis*, *A. veronii* and *A. hydrophila*. The enrichment of aerolysin (100%), T3SS (63.6%) and StcE in wound pus therefore most likely mirrors species composition rather than genuine niche adaptation, a view supported by the absence of source-associated differences in VF load (*p* = 0.277). Taken together with the species–site association described above, these data indicate that species identity—rather than isolation site per se—is the primary correlate of both the clinical distribution and the pathogenic potential of *Aeromonas* isolates.

### Limitations

This study has several limitations. First, it was conducted at a single center with a modest sample size, and the distribution of specimen types may partly reflect local sampling practices rather than true infection epidemiology. Moreover, detailed clinical information (diagnoses, comorbidities, antimicrobial treatment and outcomes) was not uniformly documented in the laboratory information system and, under the approved retrospective protocol, could not be linked to the archived isolates in a standardized manner; the analyses were therefore restricted to the microbiological and genomic level, so isolation of *Aeromonas* from a given site cannot be equated with proven infection, particularly for non-sterile sites such as sputum. Prospective studies with systematically linked clinical data will be required to assess how the species-structured resistance and virulence patterns described here translate into clinical presentation and outcome. Second, genotype–phenotype correlations were assessed at the drug-class level, which may dilute agent-specific associations, and no expression or functional assays were performed, so the contribution of inducible or silent resistance or virulence genes could not be quantified; in addition, the short-read assemblies did not resolve whether the *bla_NDM-1_* element is plasmid-borne or chromosomally integrated. Third, carriage of a gene does not imply expression, and transcript-level or proteomic validation is warranted. Fourth, our virulence analysis is limited to the prediction of putative virulence factor genes and does not demonstrate their expression or phenotypic activity (e.g., haemolysis on blood agar or cytotoxicity in cell culture); dedicated functional assays will be required to confirm the contribution of individual factors. Finally, *A. media* (*n* = 2) and *A. trota* (*n* = 1) were too few for robust inference, and the species–source confounding limits the extrapolation of source-related findings. Larger multicenter studies integrating clinical syndromes and balanced species–source compositions are warranted to confirm and extend the associations observed here.

## Conclusion

Our data delineate the clinical ecology, resistome and virulome of 74 extra-intestinal *Aeromonas* isolates from a Chinese tertiary hospital: a genetically highly diverse population dominated by *A. caviae*, *A. veronii* and *A. dhakensis*, with a strong predilection for the biliary tract of elderly male patients and a statistically significant association between species and isolation site. The predictive value of WGS-based resistome analysis in *Aeromonas* is highly drug-class dependent—excellent for trimethoprim/sulfamethoxazole, substantial for fluoroquinolones, and poor for β-lactams because of the ubiquitous intrinsic β-lactamase background—and the detection of *bla_NDM-1_* highlights the genus as a reservoir of clinically critical resistance determinants. Putative virulence profiles are strongly stratified by species: *A. dhakensis* and *A. hydrophila* harbor richer accessory virulence determinants (aerolysin, ExoA, MARTX/RtxA, T6SS) and thus greater potential virulence, whereas *A. caviae* has the sparsest virulome. Genome-based species identification should therefore be the primary basis for assessing the clinical risk posed by *Aeromonas* isolates. As the predictive performance of resistome analysis proved to be highly drug-class dependent in this collection, genotypic predictions need to be interpreted together with phenotypic susceptibility results, particularly for β-lactams.

## Data Availability

The data presented in the study are deposited in the NCBI, accession number PRJNA1494748.
