# Supplementary material for: Species distribution, antimicrobial resistance, and putative virulence factor profiles of Aeromonas isolates from extra-intestinal clinical specimens: a whole-genome sequencing study
Source: Front Microbiol. 2026 Sep 23;17:1952096. doi: 10.3389/fmicb.2026.1952096 (PMC13642587; doi:10.3389/fmicb.2026.1952096)
Supplement: Supplementary file 1 [file Table_1.DOCX]

**Supplementary tables**

**Supplementary Table S1.** Characteristics of the 74 clinical *Aeromonas* isolates included in this study. ST, sequence type (MLST). MALDI-TOF MS, matrix-assisted laser desorption/ionization time-of-flight mass spectrometry (VITEK MS, IVD knowledge base V3.2, bioMérieux); identifications are shown as reported by the instrument.

| **Strain** | **Species** | **Source of isolation** | **Gender** | **Age (years)** | **ST (MLST)** | **MALDI-TOF MS identification** |
| --- | --- | --- | --- | --- | --- | --- |
| A12525 | A.caviae | Urine | Female | 80 | 3971 | A. caviae |
| A12527 | A.hydrophila | Urine | Male | 78 | 367 | A. hydrophila |
| A12528 | A.veronii | Wound pus | Male | 56 | 425 | A. hydrophila |
| A12529 | A.veronii | Bile | Male | 80 | 1247 | A. hydrophila |
| A12530 | A.hydrophila | Wound pus | Male | 34 | 363 | A. hydrophila |
| A12532 | A.caviae | Bile | Male | 65 | 4002 | A. hydrophila |
| A12533 | A.veronii | Bile | Female | 56 | 4003 | A. hydrophila |
| A12534 | A.caviae | Bile | Female | 69 | 3973 | A. caviae |
| A12535 | A.trota | Bile | Female | 55 | 4004 | A. sobria |
| A12537 | A.caviae | Bile | Female | 36 | 3974 | A. caviae |
| A12539 | A.veronii | Bile | Female | 71 | 90 | A. hydrophila |
| A12540 | A.caviae | Sputum | Male | 75 | 4005 | A. caviae |
| A12541 | A.veronii | Blood | Male | 67 | 3975 | A. hydrophila |
| A12542 | A.dhakensis | Wound pus | Male | 58 | 3976 | A. caviae |
| A12557 | A.hydrophila | Bile | Male | 78 | 3902 | A. hydrophila |
| A12559 | A.caviae | Sputum | Male | 71 | 3977 | A. caviae |
| A12560 | A.dhakensis | Bile | Female | 50 | 4006 | A. hydrophila |
| A12561 | A.caviae | Sputum | Female | 66 | 3978 | A. hydrophila |
| A12562 | A.hydrophila | Bile | Male | 69 | 721 | A. hydrophila |
| A12563 | A.hydrophila | Urine | Male | 89 | 721 | A. hydrophila |
| A12565 | A.dhakensis | Urine | Male | 60 | 533 | A. hydrophila |
| A12566 | A.dhakensis | Wound pus | Male | 61 | 3979 | A. jandaei |
| A12567 | A.caviae | Urine | Male | 66 | 3317 | A. punctata |
| A12570 | A.dhakensis | Bile | Male | 81 | 3980 | A. jandaei |
| A12571 | A.caviae | Blood | Male | 50 | 2424 | A. caviae |
| A12572 | A.caviae | Bile | Female | 78 | 4007 | A. caviae |
| A12573 | A.caviae | Sputum | Male | 79 | 3544 | A. caviae |
| A12574 | A.caviae | Sputum | Male | 67 | 3544 | A. caviae |
| A12576 | A.media | Sputum | Male | 75 | 4008 | A. hydrophila |
| A12577 | A.dhakensis | Bile | Male | 50 | 4009 | A. hydrophila |
| A12578 | A.dhakensis | Bile | Male | 72 | 696 | A. veronii |
| A12580 | A.dhakensis | Blood | Female | 70 | 795 | A. caviae |
| A12581 | A.veronii | Bile | Male | 66 | 1005 | A. sobria |
| A12582 | A.media | Sputum | Male | 64 | 4010 | A. media |
| A12587 | A.dhakensis | Bile | Male | 55 | 710 | A. hydrophila |
| A12590 | A.hydrophila | Urine | Male | 89 | 1177 | A. hydrophila |
| A12592 | A.veronii | Wound pus | Female | 66 | 911 | A. veronii |
| A12593 | A.veronii | Wound pus | Female | 59 | 3558 | A. sobria |
| A12594 | A.veronii | Bile | Male | 82 | 4011 | Aeromonas sp. |
| A12595 | A.caviae | Sputum | Male | 71 | 3981 | A. caviae |
| A12596 | A.dhakensis | Bile | Male | 67 | 534 | A. sobria |
| A12597 | A.dhakensis | Bile | Male | 76 | 3982 | A. sobria |
| A14504 | A.caviae | Sputum | Male | 88 | 4012 | A. caviae |
| A14506 | A.veronii | Wound pus | Male | 71 | 3983 | A. veronii |
| A14509 | A.caviae | Urine | Male | 83 | 4013 | A. caviae |
| A14511 | A.caviae | Bile | Male | 71 | 3984 | A. caviae |
| A14515 | A.caviae | Bile | Female | 58 | 4014 | A. caviae |
| A14516 | A.dhakensis | Wound pus | Male | 37 | 3985 | A. hydrophila |
| A14517 | A.dhakensis | Bile | Female | 66 | 111 | A. hydrophila |
| A14520 | A.veronii | Bile | Female | 67 | 4015 | A. hydrophila |
| A14523 | A.hydrophila | Bile | Male | 69 | 1177 | A. hydrophila |
| A14524 | A.dhakensis | Wound pus | Male | 41 | 540 | A. caviae |
| A14525 | A.veronii | Bile | Male | 72 | 4016 | A. sobria |
| A14526 | A.veronii | Bile | Female | 70 | 4017 | A. sobria |
| A14527 | A.caviae | Bile | Male | 72 | 1056 | A. caviae |
| A14528 | A.caviae | Sputum | Female | 78 | 4022 | A. caviae |
| A14532 | A.dhakensis | Wound pus | Male | 17 | 311 | A. sobria |
| A14534 | A.caviae | Bile | Male | 92 | 3629 | A. caviae |
| A14536 | A.dhakensis | Bile | Male | 69 | 3986 | A. hydrophila |
| A14537 | A.caviae | Sputum | Male | 71 | 3987 | A. punctata |
| A14541 | A.caviae | Bile | Female | 53 | 1825 | A. caviae |
| A14542 | A.veronii | Bile | Male | 72 | 3988 | A. hydrophila |
| A14543 | A.veronii | Blood | Male | 78 | 512 | A. sobria |
| A14545 | A.veronii | Bile | Female | 66 | 3989 | A. veronii |
| A14546 | A.veronii | Blood | Male | 79 | 4019 | A. sobria |
| A71317 | A.hydrophila | Blood | Male | 73 | 134 | A. hydrophila |
| A71328 | A.hydrophila | Wound pus | Female | 60 | 325 | A. hydrophila |
| A71339 | A.hydrophila | Urine | Male | 85 | 2524 | A. hydrophila |
| A71431 | A.dhakensis | Bile | Female | 56 | 3636 | A. hydrophila |
| A71442 | A.caviae | Bile | Male | 55 | 3990 | A. hydrophila |
| A71453 | A.dhakensis | Bile | Male | 76 | 3684 | A. hydrophila |
| A71474 | A.veronii | Bile | Female | 65 | 164 | A. veronii |
| A71485 | A.caviae | Bile | Female | 72 | 4020 | A. hydrophila |
| A71506 | A.veronii | Bile | Male | 72 | 4021 | A. hydrophila |

**Supplementary Table S2.** Antimicrobial resistance genes detected in one or two of the 74 *Aeromonas* isolates (n = 44 genes), ranked by detection rate.

| **Resistance gene** | **Drug class(es) (CARD)** | **Gene family** | **Isolates, n** | **Detection rate, %** |
| --- | --- | --- | --- | --- |
| *AAC(3)-IId* | aminoglycoside antibiotic | AAC(3) | 2 | 2.7 |
| *CTX-M-3* | cephalosporin; penicillin beta-lactam | CTX-M beta-lactamase | 2 | 2.7 |
| *CepS* | cephalosporin | CepS beta-lactamase | 2 | 2.7 |
| *AQU-1* | cephalosporin | AQU beta-lactamase | 2 | 2.7 |
| *BRP(MBL)* | glycopeptide antibiotic | Bleomycin resistant protein | 2 | 2.7 |
| *OXA-1154* | penicillin beta-lactam | OXA beta-lactamase | 2 | 2.7 |
| *OXA-726* | penicillin beta-lactam | OXA beta-lactamase; OXA-12-like beta-lactamase | 2 | 2.7 |
| *OXA-1160* | penicillin beta-lactam | OXA beta-lactamase | 2 | 2.7 |
| *MOX-9* | cephalosporin; penicillin beta-lactam | MOX beta-lactamase | 2 | 2.7 |
| *OXA-10* | cephalosporin; penicillin beta-lactam | OXA beta-lactamase; OXA-10-like beta-lactamase | 2 | 2.7 |
| *MOX-12* | cephalosporin; penicillin beta-lactam | MOX beta-lactamase | 2 | 2.7 |
| *mcr-3.27* | peptide antibiotic | MCR phosphoethanolamine transferase | 2 | 2.7 |
| *dfrA14* | diaminopyrimidine antibiotic | trimethoprim resistant dihydrofolate reductase dfr | 2 | 2.7 |
| *OXA-959* | penicillin beta-lactam | OXA beta-lactamase; OXA-12-like beta-lactamase | 2 | 2.7 |
| *APH(3')-VIa* | aminoglycoside antibiotic | APH(3') | 1 | 1.4 |
| *APH(3'')-Ib* | aminoglycoside antibiotic | APH(3'') | 1 | 1.4 |
| *AAC(6')-Ib4* | aminoglycoside antibiotic | AAC(6') | 1 | 1.4 |
| *AAC(6')-Ia* | aminoglycoside antibiotic | AAC(6') | 1 | 1.4 |
| *ANT(2'')-Ia* | aminoglycoside antibiotic | ANT(2'') | 1 | 1.4 |
| *AQU-3* | cephalosporin | AQU beta-lactamase | 1 | 1.4 |
| *EreA2* | macrolide antibiotic | macrolide esterase | 1 | 1.4 |
| *OXA-1* | cephalosporin; penicillin beta-lactam | OXA beta-lactamase; OXA-1-like beta-lactamase | 1 | 1.4 |
| *OXA-1037* | penicillin beta-lactam | OXA beta-lactamase | 1 | 1.4 |
| *OXA-1143* | penicillin beta-lactam | OXA beta-lactamase | 1 | 1.4 |
| *OXA-1156* | penicillin beta-lactam | OXA beta-lactamase | 1 | 1.4 |
| *OXA-1162* | penicillin beta-lactam | OXA beta-lactamase; OXA-12-like beta-lactamase | 1 | 1.4 |
| *mcr-3.3* | peptide antibiotic | MCR phosphoethanolamine transferase | 1 | 1.4 |
| *MOX-21* | cephalosporin; penicillin beta-lactam | MOX beta-lactamase | 1 | 1.4 |
| *OXA-2* | cephalosporin; penicillin beta-lactam | OXA beta-lactamase; OXA-2-like beta-lactamase | 1 | 1.4 |
| *OXA-12* | penicillin beta-lactam | OXA beta-lactamase; OXA-12-like beta-lactamase | 1 | 1.4 |
| *PME-1* | carbapenem | PME beta-lactamase | 1 | 1.4 |
| *OXA-917* | carbapenem; penicillin beta-lactam | OXA beta-lactamase; OXA-427-like beta-lactamase | 1 | 1.4 |
| *TRU-1* | cephalosporin; penicillin beta-lactam | TRU beta-lactamase | 1 | 1.4 |
| *VEB-1* | monobactam; cephalosporin | VEB beta-lactamase | 1 | 1.4 |
| *Vibrio anguillarum* chloramphenicol acetyltransferase | phenicol antibiotic | chloramphenicol acetyltransferase (CAT) | 1 | 1.4 |
| *qnrVC1* | fluoroquinolone antibiotic | quinolone resistance protein (qnr) | 1 | 1.4 |
| *aadA16* | aminoglycoside antibiotic | ANT(3'') | 1 | 1.4 |
| *arr-2* | rifamycin antibiotic | rifampin ADP-ribosyltransferase (Arr) | 1 | 1.4 |
| *cmlA5* | phenicol antibiotic | major facilitator superfamily (MFS) antibiotic efflux pump | 1 | 1.4 |
| *arr-3* | rifamycin antibiotic | rifampin ADP-ribosyltransferase (Arr) | 1 | 1.4 |
| *cmlA9* | phenicol antibiotic | major facilitator superfamily (MFS) antibiotic efflux pump | 1 | 1.4 |
| *cphA7* | carbapenem | CphA beta-lactamase | 1 | 1.4 |
| *dfrA15* | diaminopyrimidine antibiotic | trimethoprim resistant dihydrofolate reductase dfr | 1 | 1.4 |
| *qacL* | disinfecting agents and antiseptics | small multidrug resistance (SMR) antibiotic efflux pump | 1 | 1.4 |

Supplementary Table S3. Species-stratified resistance rates of the 74 *Aeromonas* isolates against the 11 tested agents. Values are the number of resistant isolates (percentage within species). Rates are descriptive only and were not subjected to inferential testing; *A. media* (n = 2) and *A. trota* (n = 1) are shown for completeness. TZP, piperacillin/tazobactam; CAZ, ceftazidime; SCF, cefoperazone/sulbactam; FEP, cefepime; ATM, aztreonam; IPM, imipenem; MEM, meropenem; CIP, ciprofloxacin; LEV, levofloxacin; AMK, amikacin; SXT, trimethoprim/sulfamethoxazole.

| **Species** | **TZP** | **CAZ** | **SCF** | **FEP** | **ATM** | **IPM** | **MEM** | **CIP** | **LEV** | **AMK** | **SXT** |
| --- | --- | --- | --- | --- | --- | --- | --- | --- | --- | --- | --- |
| *A. caviae* (n = 24) | 4 (16.7) | 4 (16.7) | 4 (16.7) | 3 (12.5) | 1 (4.2) | 1 (4.2) | 2 (8.3) | 3 (12.5) | 3 (12.5) | 0 (0.0) | 5 (20.8) |
| *A. veronii* (n = 19) | 8 (42.1) | 1 (5.3) | 0 (0.0) | 0 (0.0) | 1 (5.3) | 4 (21.1) | 3 (15.8) | 2 (10.5) | 1 (5.3) | 1 (5.3) | 3 (15.8) |
| *A. dhakensis* (n = 18) | 7 (38.9) | 4 (22.2) | 5 (27.8) | 1 (5.6) | 0 (0.0) | 4 (22.2) | 2 (11.1) | 0 (0.0) | 0 (0.0) | 0 (0.0) | 3 (16.7) |
| *A. hydrophila* (n = 10) | 3 (30.0) | 3 (30.0) | 1 (10.0) | 1 (10.0) | 1 (10.0) | 3 (30.0) | 1 (10.0) | 1 (10.0) | 0 (0.0) | 0 (0.0) | 3 (30.0) |
| *A. media* (n = 2) | 0 (0.0) | 0 (0.0) | 0 (0.0) | 0 (0.0) | 0 (0.0) | 0 (0.0) | 0 (0.0) | 0 (0.0) | 0 (0.0) | 0 (0.0) | 0 (0.0) |
| *A. trota* (n = 1) | 0 (0.0) | 0 (0.0) | 0 (0.0) | 0 (0.0) | 0 (0.0) | 0 (0.0) | 0 (0.0) | 0 (0.0) | 0 (0.0) | 0 (0.0) | 0 (0.0) |
| **Total (n = 74)** | **22 (29.7)** | **12 (16.2)** | **10 (13.5)** | **5 (6.8)** | **3 (4.1)** | **12 (16.2)** | **8 (10.8)** | **6 (8.1)** | **4 (5.4)** | **1 (1.4)** | **14 (18.9)** |

Supplementary Table S4. Per-isolate antimicrobial susceptibility profiles of the 74 *Aeromonas* isolates against the 11 tested agents (VITEK 2 automated broth microdilution; S, susceptible; I, intermediate; R, resistant; interpreted according to CLSI guideline M45). TZP, piperacillin/tazobactam; CAZ, ceftazidime; SCF, cefoperazone/sulbactam; FEP, cefepime; ATM, aztreonam; IPM, imipenem; MEM, meropenem; CIP, ciprofloxacin; LEV, levofloxacin; AMK, amikacin; SXT, trimethoprim/sulfamethoxazole.

| **Isolate** | **TZP** | **CAZ** | **SCF** | **FEP** | **ATM** | **IPM** | **MEM** | **CIP** | **LEV** | **AMK** | **SXT** |
| --- | --- | --- | --- | --- | --- | --- | --- | --- | --- | --- | --- |
| A12525 | S | S | S | S | S | S | S | S | S | S | S |
| A12527 | R | R | R | I | S | R | R | S | I | S | R |
| A12528 | R | S | S | S | S | S | S | S | S | S | S |
| A12529 | R | S | S | S | S | S | S | S | S | S | S |
| A12530 | R | S | S | S | S | S | S | S | S | S | S |
| A12532 | S | S | S | S | S | S | S | S | S | S | S |
| A12533 | S | S | S | S | S | S | S | S | S | S | S |
| A12534 | S | S | S | S | S | S | S | S | S | S | S |
| A12535 | S | S | S | S | S | S | S | S | S | S | S |
| A12537 | S | S | S | S | S | S | S | S | S | S | S |
| A12539 | R | S | S | S | R | R | R | S | S | S | S |
| A12540 | S | S | S | S | S | S | S | S | S | S | S |
| A12541 | R | S | S | S | S | S | S | S | S | S | S |
| A12542 | R | S | S | S | S | S | S | S | S | S | S |
| A12557 | R | S | I | S | S | R | S | S | S | S | S |
| A12559 | S | S | S | S | S | S | S | S | S | S | S |
| A12560 | R | R | R | S | S | R | S | S | S | S | S |
| A12561 | S | S | S | S | S | S | S | S | S | S | R |
| A12562 | S | S | S | S | S | S | S | S | S | S | S |
| A12563 | S | S | S | S | S | S | S | S | S | S | S |
| A12565 | S | S | S | S | S | S | S | S | S | S | S |
| A12566 | S | S | S | S | S | R | R | S | S | S | S |
| A12567 | R | R | R | R | R | S | S | R | R | S | R |
| A12570 | R | R | R | I | I | R | R | S | I | S | S |
| A12571 | S | S | I | S | S | S | S | S | I | S | S |
| A12572 | S | S | S | S | S | S | S | I | I | S | S |
| A12573 | S | S | S | S | S | S | S | I | S | S | S |
| A12574 | S | S | S | S | S | S | S | I | S | S | S |
| A12576 | S | S | S | S | S | S | S | S | S | S | S |
| A12577 | S | S | S | S | S | S | S | S | S | S | S |
| A12578 | S | S | S | S | S | S | S | S | S | S | S |
| A12580 | S | S | S | S | S | S | S | I | I | S | R |
| A12581 | S | S | S | S | S | S | S | S | S | S | S |
| A12582 | S | S | S | S | S | S | S | S | S | S | S |
| A12587 | R | R | R | S | S | S | S | S | S | S | S |
| A12590 | S | R | S | S | S | S | S | S | S | S | R |
| A12592 | S | S | S | S | S | S | S | S | S | S | S |
| A12593 | S | S | S | S | S | S | S | S | S | S | S |
| A12594 | S | S | S | S | S | R | R | S | S | S | S |
| A12595 | S | S | S | S | S | S | S | S | I | S | S |
| A12596 | R | S | R | S | S | S | S | S | S | S | R |
| A12597 | S | S | S | S | S | S | S | S | S | S | S |
| A14504 | R | R | R | I | S | R | R | R | R | S | R |
| A14506 | S | S | S | S | S | R | R | S | S | S | R |
| A14509 | R | R | R | R | S | S | R | R | R | S | R |
| A14511 | S | S | S | S | S | S | S | S | S | S | S |
| A14515 | S | S | S | S | S | S | S | S | S | S | S |
| A14516 | S | S | S | S | S | S | S | S | S | S | S |
| A14517 | S | S | S | S | S | S | S | S | S | S | R |
| A14520 | S | S | S | S | S | S | S | S | S | S | S |
| A14523 | S | R | S | R | R | S | S | S | S | S | R |
| A14524 | R | S | S | S | S | S | S | S | S | S | S |
| A14525 | S | S | S | S | S | S | S | S | S | S | S |
| A14526 | R | S | S | S | S | S | S | S | S | S | S |
| A14527 | S | S | S | S | S | S | S | S | S | S | S |
| A14528 | S | S | S | S | S | S | S | S | I | S | S |
| A14532 | S | S | S | S | S | S | S | S | S | S | S |
| A14534 | S | S | S | S | S | S | S | S | S | S | S |
| A14536 | R | R | R | R | S | S | S | S | S | S | S |
| A14537 | S | S | S | S | S | S | S | S | S | S | S |
| A14541 | S | S | S | S | S | S | S | S | S | S | S |
| A14542 | R | S | S | S | S | I | S | S | S | S | S |
| A14543 | R | S | S | S | S | I | S | S | S | S | S |
| A14545 | S | R | S | S | I | R | S | S | S | R | R |
| A14546 | R | S | S | S | S | I | S | R | R | S | S |
| A71317 | S | S | S | S | S | S | S | S | S | S | S |
| A71328 | S | S | S | S | S | S | S | S | S | S | S |
| A71339 | S | S | S | S | S | R | I | R | I | S | S |
| A71431 | S | S | S | S | S | R | S | S | S | S | S |
| A71442 | S | S | I | S | S | S | S | S | S | S | R |
| A71453 | S | S | S | S | S | S | S | S | S | S | S |
| A71474 | S | S | S | S | I | S | S | S | S | S | S |
| A71485 | R | R | R | R | S | S | S | S | S | S | S |
| A71506 | S | S | S | S | S | S | S | R | I | S | R |
